# Outlines of Botany

**Published:** 1835-04-01

**Authors:** 


### OUTLINES OF BOTANY, &c. By *Gilbert T. Burnett*, F.L.S. London: 2 Vols. 8vo. pp. 1190.

IT is a problem of the highest philosophical interest, but one which is abstruse and difficult in the extreme, to arrange the almost countless productions of the vegetable kingdom according to some natural affinities, which shall be at once authentic, unvarying, and of easy recognition. The master mind of the great Swedish botanist certainly achieved a more distinguished success in this arduous undertaking than had been done by any previous to his time; and almost all his successors have paid the homage due to his transcendent talents, by adopting, with varying modifications indeed, his views as a groundwork of the classifications which they have proposed. The division of plants into such as are flower-bearing, and, therefore, seed-bearing; and such as are destitute of inflorescence, and are, therefore, seedless, was proposed first, we believe, by Ray, the old English botanist. The former are grouped together by Linnæus under the general denomination of phænogamous, *i. e.* having, to use the metaphorical language of his system, an obvious and distinct marriage or mode of generation. They are all provided with the essential parts of a flower, viz. the stamens and pistils (which it is well known are regarded as the male and female organs), either on one plant, or on different plants of the same species. These two parts are necessary for the formation of a genuine seed—we add the word "genuine" to mark the difference between the product of the bisexual vegetable union, just noticed, and the falsely-called seeds of ferns, mosses, mushrooms, &c. which the botanist distinguishes by the name of spores, or sporules. The former, the genuine seed, consists of several parts which are generally and easily demonstrable, viz. a minute body called the embryo or plantule, a reservoir of food laid up for its support during the infantile period of its growth, which bag of starch is called the albumen, and the cotyledons, or seed-lobes, which are closely attached to the embryo, and either ascend out of the ground, and perform for a while the office of leaves, or remain buried till they gradually decay. The latter, or spores, are destitute of radicle and plumule (the two parts of the embryo), and also of any distinct cotyledon; they have the power of striking root indifferently from any part of their surface. They have, therefore, the character rather of buds than of seeds; and it is a curious coincidence, that many of the "spore-bearing" plants are reproductive in another way, viz. by portions (frustula or gonidia) becoming detached from the parent stem, and forming independent and self-existing plants. The second division of the vegetable kingdom includes all plants which are called cryptogamous, or whose marriage or mode of generation is hidden or unknown; they are destitute of flowers, and, therefore, of seeds, and are propagated either by spores or by frustules.

From the preceding brief details, it is obvious that the terms phænogamous and cryptogamous are equivalent or nearly so, to the more recent terms, cotyledonous and acotyledonous (and these, again, are synonymous with seed-bearing and seedless), which have of late been very generally adopted. This twofold division is the first step in the synthetic classification of vegetables; the second is the almost equally important splitting of the first class into such plants as have seeds provided with one cotyledon, or seed-lobe, and hence called mono-cotyledonous, or uni-lobate; and into such as have two cotyledons, and are, therefore, called dicotyledonous, or bilobate plants.

The tripartite division into acotyledonous, monocotyledonous, and dicotyledonous orders, apparently so arbitrary and gratuitous, is found to harmonize with, and also to indicate, at least in many very important respects, other natural affinities which may be said to establish strong family resemblances among certain extensive groups of the vegetable world.

If we examine the structure or tissue of those plants which belong to the class cryptogamia, and which are, therefore seedless, or acotyledonous, it will be found that in all, with the exception of the ferns, it is uniformly, and throughout every part, truly cellular or vesicular, *i. e.* composed solely of an infinitude of cells or cellules, and presenting no traces of vessels or lengthened tubes. Hence the epithet "cellulares, or cellulosæ," has been applied to this large section of the cryptogamic class. In addition to this structural difference, it is said that the tegumentary covering of these plants differs from true cuticle, in being scarcely distinguishable from the cellular substance which it incloses, and in being destitute of stomata, (which are generally signs of the presence of tubular vessels,) that their leaves, when present, are without a pleuro-phyl, or skeleton; and that their stems, even when apparently endogenous, are unstratified and homogeneous. Although these definite characters are on the whole strictly and exclusively applicable to the three earliest orders of the cryptogamic class, viz. the algæ, fungi, and musci, it must be confessed that certain exceptions have been pointed out to their universal truth by some eminent botanists. Thus the aquatic tribe of the charales, including the two genera, chara and nitella, vulgarly called stoneworts, exhibits "a structure so peculiar, that every attempt to associate them with other orders has failed; and even the propriety of their location among the mosses is not unquestionable. The cellules are so much extended that they must be regarded as tubuli, and they thus mark the transition of cells into tribes, or the change of vesicles into vessels, commonly so called." Again, in sphagnum, and one or two other instances, it is asserted that tubular vessels have been found, as well as fibro-membranous cells, which are the rudiments of spiral tubes. Hooker and Mirbel have discovered also in some few cases perforations in the leaves, and these they have proved to be genuine stomata, which were previously denied to be present in any of the mosses, or their allies, and indeed in any plants lower in the scale than the ferns.

If the objections now adduced be found on subsequent enquiries to be authentic, the propriety of the epithet "cellulares," or "cellulosæ," as characteristic of the three first orders of cryptogamic plants, may be fairly impugned on this ground alone; but there are other more weighty arguments, which recent botanical discoveries have most unexpectedly afforded.

There are certain splendid oriental parasitical plants, and several others, natives both of Europe and of America, which exhibit a most distinct inflorescence, and yet which are strictly "cellular, or almost vascular," in their structure. Here then the deductions from vegetable anatomy are at variance with the laws or general facts (for these are synonymous terms) of vegetable physiology, and therefore with the bases of a natural classification of plants. We see plants, which on the one hand are provided with gigantic flowers, of a yard and upwards across the disk, and which, on the other hand, by their destitution of tubular vessels, and their fungoid characters, (of having no leaves, and being parasitic in their growth,) shew their close affinity to the lower mushroom sections; thus seeming to form the descending links which connect the highest with the lowest grades of vegetable development.

It is however but proper to mention that, within the last year, Dr. Brown, who, when he published his admirable monograph on the Rafflesia Arnoldi, asserted its exclusively cellular structure, has communicated to the Linnæan Society the results of some more recent observations, which have led him to modify considerably his former opinion. He has found tubular vessels in the bud-scales of the Rafflesia; but "they were very few in number, and not only spare, but also oftentimes imperfect." The fruit likewise he describes as being not quite so simple as had been previously supposed; for although "the seeds consist of a soft and nearly homogeneous mass, albumen and embryo are distinguishable, and hence, although spore-like, they are not veritable spores." But while we admit the correctness of these remarks, and whoever is acquainted with the pre-eminent talents of Dr. Brown, will at once be prepared to assent, the conclusion is still forced upon us that the Selanthi, including the cytini, (the hypocist of the ancients,) cynomorium or fungus melitensis, (Maltese mushroom,) brugmansia, rafflesia, &c. are essentially of a cellular texture, and approximate to some cryptogamic plants by being destitute, or nearly so, of tubular vessels; by having no leaves, the foliage consisting of brown or colourless scales; and by their seeds often consisting of a mass of homogeneous grumous matter.

Whoever has followed us attentively through the preceding page, will now be able to appreciate the motives which have induced Professor Burnett to reject the nomenclature of Decandolle and other botanists, who have associated all cryptogamic plants under the general denomination of "cellulares," or "cellulosæ." Equally potent objections may be urged against employing the epithet acotyledonous as the title of the first great division of the vegetable kingdom; for if we admit this, we shall be necessitated to groupe together plants which differ very much in their essential attributes; and therefore to dissociate some from the natural groupes or families to which they evidently belong;—we allude particularly to the curious tribe of ferns which, although cryptogamic and acotyledonous, are nevertheless well known to bear so close a resemblance to the palms, that almost every botanist agrees that with these they ought to be classed in a natural arrangement. Influenced, no doubt, by these arguments, Professor Burnett has proposed to substitute the term "myc-affines," anglice "moss-allies," as a general or family name for the first great division of the vegetable kingdom, in which are included the algæ, fungi, and musci; those plants which he has defined as "cellular, flowerless, seedless, and propagated by spores, sporidia, or pustules." By employing an arbitrary epithet, which has no reference to any hypothetical affinity in the anatomy or physiology of the plants, he avoids the objections, which we have shewn, may be urged against the terms "cellulares" and "acotyledones." We could have wished, however, that he had been more fortunate in his philological selection. A Greek noun and a Latin adjective are reluctantly wedded together, to form a most inharmonious compound. The remarks, however, of the ingenious author on this subject at page 43 deserve an attentive perusal, and may perhaps reconcile the reader to the cacophony of the epithet.

Having thus explained the principles on which the first great section of the ternary classification of plants is founded, and mentioned the title which our author proposes to affix to it, our readers will experience but little difficulty in appreciating the distinctive characters of the remaining two sections, denominated in the present work "termaffines" and "crescaffines," (the meaning of which terms, analogous to the preceding one "mycaffines," will be apparent immediately). The plants of both sections have this important feature in common—that their tissue or structure consists not of cells alone, as in the families of the preceding section, but of tubes or vessels and of cells, more or less irregularly connected together. They are all, therefore, "cellulo-vascular," or, in the language of Decandolle, simply "vascular." So far they agree; but a most important difference in the arrangement of the cells and tubes, and in several other equally obvious and distinctive characters, exists in the two great sections of vascular plants. In the first section, the tubes and cells are interblended in one mass; and there is no distinction of parts for the ascent and descent of the sap. The bark cannot be distinguished from the wood, nor any layer of new or sap-wood from another layer of an older formation. Hence such plants have been called " unstratified;" and from the circumstance of the annually new fibres, by which the vital actions of the plant are performed, being always deposited centrally within, or interior to the older ones, (which are therefore gradually pushed outwards to the surface, so that the external parts are always the oldest and hardest,) they have also received the appellation of " endogenæ," or " inside-growers."

In the second section of vascular plants the arrangement of the cells and tubes is much more regular ; and instead of the unstratified texture of the "endogenæ," we find a beautiful series of concentric woody circles or layers surrounding a central pith, and surrounded by an external series of different form, and size, and thickness, which constitute the bark.

It is well known that the concentric layers, now mentioned, are indicative of regular and annual successive deposits, each new one being exterior to those already formed. Such a growth may therefore be truly said to take place outwardly, and hence all plants in which the structure now mentioned is found, have been denominated "exogenæ" or outside growers. Besides this essential difference of structure, we may state that, the external form of the trunk is conical and not cylindrical, as in the endogenæ; it is branched not simple; the leaves are articulated with the stems or branches; the embryo consists of two seed-lobes, and the radicle is naked. Such are the two great sections or divisions of the class " vasculares," a class which includes the whole vegetable kingdom, with the exception of the three orders, algæ, fungi, and musci, which we have already shewn to be cellular in their structure. The ferns (an acotyledonous tribe of plants) and all the monocotyledones, such as the grasses, the rushes, the liliaceæ, the lordly palms, and the graceful musæ, belong to the " endogenæ," while the dicotyledones, commencing with the very natural groupe of the pines and coniferæ, and comprehending the countless varieties of plants, whose seeds are invested with a peculiar covering called a pericarp, (known commonly as the fruit,) and which were therefore associated by the older botanists under the general terms of fruges, eucarpæ, or, according to the Linnæan language of herbs and trees, are all grouped together, as being strictly " exogenous" in their growth. It requires however to be stated, that some apparent exceptions to the universality of the doctrine inculcated in the two epithets " endogenæ" and " exogenæ," have been recognized by botanical physiologists. Thus the dracæna, or dragon's-blood tree, one of the liliaceæ, "the structure of whose unstratified stem would decidedly place it amongst the endogenæ, even if no reference were made to its foliage, and monocotyledonous seeds, is still any thing strictly endogenous, *i. e.* inside growing: its stem is branched, is not cylindrical, and increases in girth, as it increases in age." Again, the pandanus among the typhinæ, the branching asphodelaceæ, and also the smilaceæ, &c. having a vein-like reticulation of their leaves, &c. may be adduced as examples of deviation in various ways from the strict and leading characters of the groupe.

Our readers will now be prepared to understand the motives which have led our author to propose two new terms, in the place of those introduced by Decandolle, viz. endogenæ and exogenæ. All endogenous plants, from the very mode of their growth, are necessarily of very limited duration ; for, as the peripheral layer of their stems becomes firmer and harder each year, in consequence of the internally deposited formation causing a gradually increasing pressure from within outwards, a period at length arrives when this hardened outer girth becomes unyielding, and the internal space being filled up, no further deposits can take place, and the plants inevitably die.

"The term of their existence (says Prof. B.) being thus fixed during their earliest years, which the very act of growth renders more and more inevitable, and which is strengthened by their strength, may not improbably have led the ancients to apply the name 'termes' to a palm-tree, as well as to the fruit branches of other plants, when plucked, and a period put to their existence; and hence the region in which this peculiar characteristic is found to prevail may be called 'term-affines,' as indicative of this, one of the most notorious diagnostic signs."

On the other hand, the mode of growth in exogenous plants sets no necessary limits to the term of their duration. The new deposits are external to the old ones, and as there is, at the same time, an annual reproduction of bark, no constriction, such as takes place in the former class, the "term-affines," is induced,—a constriction which we have explained to be the cause of the very limited existence of these last mentioned plants.

The natural tendency, therefore, of the exogenæ is to increase, year after year, in circumference as well as in height; and the prodigious dimensions of some oaks, chesnuts, and baobabs, attest their extreme antiquity. What shall we say of trees, with trunks of 100, and even 150 feet in circumference, and whose ages are upwards of 2000 years. This tendency to mighty increase of dimensions, and to very protracted duration, constitutes a very striking characteristic of exogenous plants, the structure of which sets naturally no limit to their existence. The term "cresc-affines" has been, for these reasons, proposed by our author to designate this very extensive, and by far the most important, division of the vegetable world.

It is not our intention to discuss the appropriateness of the nomenclature which Professor Burnett has substituted for that of Decandolle and other botanists; we may fairly leave this topic to the judgment of the reader, and, if we mistake not, the verdict will be nearly unanimous. It may be allowed to be ingenious; but it is far too vague in its allusions, as well as unclassical in its composition, to be received with much favour. Fortunately, the merits of these Outlines are not suspended on so frail a thread as the fitness of any part of its scientific language. They will be found to contain a greater accumulation of curious and instructive details on botanical subjects, than any other English work with which we are acquainted.

The unwearied labour and ardent love of his favourite science displayed in every page, reflect the most distinguished credit on Professor Burnett, and deserve to be rewarded by the patronage of the public in general, and more especially of the medical profession. We might have wished that the author had, for his own sake, given a more appropriate title to this work than "Outlines of Botany"—a title which is apt to convey a very imperfect notion of its contents; for, independently of a general review of the whole vegetable world, the reader will find a more or less detailed description of almost every genus of plants, with pertinent remarks on the more curious facts of their physiology, of their medical and dietetic processes, and of their geographical and geological distribution; and wherever the subject admits of illustration by the pencil, the aid of the draughtsman has been called into very pleasing requisition. Many of the woodcuts are indeed excellent.

